# Editorial: Advances in spinal cord injury prevention during endovascular and open aortic repairs

**DOI:** 10.3389/fsurg.2026.1794723

**Published:** 2026-02-16

**Authors:** Mohamed Rahouma, Massimo Baudo, Magdy El-Sayed Ahmed

**Affiliations:** 1 Cardiothoracic Surgery Department, Weill Cornell Medical Center, New York-Presbyterian, New York, NY, United States; 2 Surgical Oncology Department, National Cancer Institute, Cairo University, Cairo, Egypt; 3 Department of Cardiac Surgery Research, Lankenau Institute for Medical Research, Wynnewood, PA, United States; 4 Cardiothoracic Surgery Department, Mayo Clinic, Jacksonville, FL, United States; 5 Department of Surgery, Faculty of Medicine, Zagazig University, Zagazig, Egypt

**Keywords:** aortic surgery, editorial, endovascular surgery, spinal cord injury, TEVAR

Neurologic injury of the spinal cord remains the most feared complication after surgery on the descending thoracic and thoracoabdominal aorta, even as endovascular approaches are increasingly adopted to treat anatomically complex disease. Despite technical progress, the reported incidence of spinal cord injury (SCI) remains clinically relevant, generally ranging from 2% to 10%, and is often associated with permanent paraplegia, with profound downstream effects on patient outcomes, including prolonged intensive care unit stays, extended hospitalization, higher short- and long-term mortality, and marked deterioration in functional status and quality of life (1, 2). SCI after thoracic or thoracoabdominal aortic interventions, whether open repair or thoracic endovascular aortic repair (TEVAR), presents as a consequence of disrupted spinal cord perfusion arising from vulnerable vascular anatomy, dependence on collateral pathways, and downstream cellular injury (3, 4). Neurological deficits that may appear immediately or after a delay and that markedly worsen prognosis and long-term function (5). Multiple spinal cord protection strategies, such as cerebrospinal fluid drainage, permissive hypertension, and optimization of spinal cord perfusion pressure, have been developed and refined (6).

This research topic in Frontiers in Cardiovascular Medicine comes to shed light on how SCI can be mitigated during aortic surgery, provide an analysis on the anatomical variability of aortic segmental arteries and describe a rare case of aortic angiosarcoma leading to paraplegia. This topic includes 4 manuscripts (2 original research papers, 1 review, and 1 case report).

The review by Torre and Pirri distinguishes SCI mechanisms by treatment modality. Open repair typically induces an ischemia–reperfusion pattern from cross-clamping, characterized by oxidative stress, excitotoxicity, barrier disruption, and inflammation, whereas endovascular repair more often produces a sustained low-flow state due to extensive segmental artery exclusion; embolization and venous congestion may occur in both. Risk is driven by the extent and level of aortic replacement or coverage, perioperative hypotension, impaired oxygen delivery, renal or peripheral vascular disease, prior aortic surgery with reduced collateral reserve, urgent presentation, and procedural complexity or duration, with TEVAR-specific risk scores aiding stratification. Prevention is framed as bundled care: preserving or restoring collateral inflow (selective left subclavian and internal iliac management), selective intercostal reattachment in open repair, and staged procedures to promote collateral adaptation, alongside emerging strategies such as segmental artery embolization preconditioning, temporary sac perfusion, and procedural sequencing to reduce pelvic or limb ischemia. Emphasis is placed on physiology-based management, including intraoperative neuromonitoring, hemodynamic and oxygen-delivery targets, cerebrospinal fluid pressure control, and distal aortic perfusion in extensive open TAAA repair. Cerebrospinal fluid drainage remains well supported in open surgery but controversial in TEVAR, given weaker evidence and non-trivial risks, underscoring the importance of rapid rescue protocols. Overall, the review points toward individualized, physiology-driven protection strategies, calling for prospective validation of standardized bundles, improved real-time monitoring, and integrated predictive models combining imaging, physiologic data, and emerging neural injury biomarkers within multicenter registries and trials.

Rosvall et al. demonstrate that sustained reduction of SCI after complex endovascular aortic repair is achievable when perioperative management is treated as a continuous physiological process rather than a procedural adjunct. Their experience shows that consistent hemodynamic optimization, rapid restoration of distal perfusion, and structured neurological monitoring can keep permanent neurologic injury rare, even in anatomically extensive or urgent repairs. Importantly, the findings question the centrality of routine cerebrospinal fluid drainage and instead emphasize early detection and prompt corrective action. The disproportionate risk observed among women, ruptured presentations, and patients with impaired renal function suggests that standardized protocols, while effective, may require further tailoring to address biologically and clinically distinct vulnerability profiles.

The anatomical investigation by Pruidze et al. provides a large, systematic description of the origin and variability of aortic segmental arteries supplying the spinal cord, structures that are critical determinants of SCI during thoracic and thoracoabdominal aortic repair. Through detailed dissection of more than 150 human cadavers, the authors demonstrate that the number, symmetry, and level of origin of segmental arteries vary far more than traditionally described in textbooks, with frequent shared origins, non-segmental branching, and marked heterogeneity in the thoracolumbar region where the artery of Adamkiewicz most often arises. These findings challenge the concept of a uniform segmental arterial pattern and highlight that individual patients may have limited collateral reserve. By providing both absolute and relative measurements of segmental artery orifices along the descending aorta, the study underscores the importance of meticulous preoperative imaging to identify high-risk anatomy, guide stent length and staging strategies, and reduce the likelihood of spinal cord ischemia during open or endovascular aortic interventions.

Finally, the case report by Li and Sun describes an exceptionally uncommon presentation of primary aortic angiosarcoma in a 76-year-old woman, where the disease manifested not only with typical tumor-related intraluminal aortic lesions and systemic embolic risk, but also with abrupt paraplegia consistent with spinal cord ischemia. Imaging showed long, irregular, minimally enhancing filling defects extending from the aortic arch through the descending aorta with involvement of branch origins, alongside a destructive left hip/sacroiliac soft-tissue mass. Biopsy of the metastatic lesion confirmed angiosarcoma by histology and endothelial-marker immunophenotype. Because direct aortic biopsy carried prohibitive embolic risk, diagnosis relied on the metastatic site plus computed tomography angiography correlation. The patient declined oncologic therapy, received anticoagulation, then rapidly developed bilateral lower-limb paralysis with loss of sensation and concomitant acute lower-limb arterial occlusion, and died shortly thereafter. The authors use the case to emphasize that aortic angiosarcoma, although rare and often mimicking thrombus/atherosclerosis, should be considered when unexplained recurrent embolic events occur, and that clinicians should monitor for both common embolic targets and unusual territories such as the spinal cord.

Collectively, the articles in this Research Topic underscore that SCI associated with aortic pathology and intervention arises from a complex and patient-specific interplay of biological responses, vascular anatomy, and procedural characteristics. Meaningful risk reduction will require a shift away from uniform protocols toward tailored strategies informed by precise anatomic assessment, continuous physiologic surveillance, and dynamic perioperative optimization.
